# Enhancing clinical data retrieval with Smart Watchers: a NiFi-based ETL pipeline for Elasticsearch queries

**DOI:** 10.1186/s12911-024-02633-w

**Published:** 2024-09-16

**Authors:** Mohammad Al-Agil, Stephen J. Obee, Vlad Dinu, James Teo, David Brawand, Piers E. M. Patten, Anwar Alhaq

**Affiliations:** 1 King’s College Hospital NHS Foundation Trust, London, UK; 2 Guy’s and St Thomas’ NHS Foundation Trust, London, UK; 3 Department of Haematology, Comprehensive Cancer Centre, King’s College London, London, UK

**Keywords:** Clinical alert systems, CogStack, ETL pipelines, Apache Nifi, Elasticsearch, Python, Automated data extraction

## Abstract

**Background:**

The aim is to develop and deploy an automated clinical alert system to enhance patient care and streamline healthcare operations. Structured and unstructured data from multiple sources are used to generate near real-time alerts for specific clinical scenarios, with an additional goal to improve clinical decision-making through accuracy and reliability.

**Methods:**

The automated clinical alert system, named Smart Watchers, was developed using Apache NiFi and Python scripts to create flexible data processing pipelines and customisable clinical alerts. A comparative analysis between Smart Watchers and the legacy Elastic Watchers was conducted to evaluate performance metrics such as accuracy, reliability, and scalability. The evaluation involved measuring the time taken for manual data extraction through the electronic patient record (EPR) front-end and comparing it with the automated data extraction process using Smart Watchers.

**Results:**

Deployment of Smart Watchers showcased a consistent time savings between 90% to 98.67% compared to manual data extraction through the EPR front-end. The results demonstrate the efficiency of Smart Watchers in automating data extraction and alert generation, significantly reducing the time required for these tasks when compared to manual methods in a scalable manner.

**Conclusions:**

The research underscores the utility of employing an automated clinical alert system, and its portability facilitated its use across multiple clinical settings. The successful implementation and positive impact of the system lay a foundation for future technological innovations in this rapidly evolving field.

## Introduction

EPR systems typically employ automated safety alerts based on structured data flags, such as laboratory results outside normal ranges or abnormalities identified by radiologists. These flags are generally assigned during data entry and rely on deterministic capture of trigger information within structured data fields. However, this approach is limited in its ability to detect abnormalities that are context-dependent or require integration of multiple data points, particularly when relevant EPR data is distributed across structured and unstructured formats or arrives asynchronously.

A considerable portion of EHR data exists in free-text format, which rarely incorporates structured flags. While it is theoretically possible to implement workflows requiring clinicians to manually assign abnormality flags, such practices are challenging to sustain due to their disruptive and potentially error-prone nature in clinical care delivery. Furthermore, the clinician entering the data may lack access to the full patient context necessary for accurate flag assignment. For example, radiologists typically operate within isolated systems (such as Radiology Information Systems or Picture Archiving Systems) and may not have visibility into other relevant patient data that could influence the interpretation of their findings [1].

The absence of comprehensive contextual information often residing in unstructured text, significantly impairs the performance of existing automated safety alert systems, necessitating extensive ongoing oversight to maintain efficacy. To address these challenges, we propose the development of an alerting system that:

- Integrates both structured and unstructured data sources.
- Employs natural language processing (NLP) techniques to extract relevant information from free-text entries.
- Dynamically adapts to incomplete, missing, or late-arriving information.
- Incorporates a feedback mechanism for continuous improvement based on clinician input.
- Minimizes additional burden on existing clinical workflows through seamless integration with current EHR systems.
- Features built-in data collection and processing capabilities to provide comprehensive context alongside each alert.

The proposed system aims to enhance clinical alert accuracy and efficiency to potentially improve patient safety outcomes and reduce the burden of manual review on healthcare providers.

### Overview of the CogStack, Nifi, Elasticsearch and Smart Watcher infrastructure at King’s College Hospital (KCH)

CogStack is a clinical analytics platform developed to extract and analyse unstructured clinical data, which helps automate many manual steps involved in clinical data gathering and patient monitoring [2]. It harnesses advanced NLP techniques and state-of-the-art data integration and storage for rapid information retrieval. The CogStack instance at KCH is one of the longest-running iterations since its establishment for the 100 k genome project in 2018. We will explore the key components of CogStack (Elasticsearch, Kibana and Apache Nifi) and the Smart Watcher system.

Elasticsearch is a highly scalable, search and analytics engine that enables the storage, searching, and analysis of large volumes of structured and unstructured data in near real-time [3]. Data is organised into indexes consisting of records, where each record represents a single document. These records are composed of fields, which are key-value pairs that contains the actual data. Fields could consist of various data types, such as text, numeric, date, or geospatial, and can be used for searching using Lucene syntax, filtering, and aggregating the data. Kibana is an open-source data visualisation and exploration tool that is specifically designed to work with Elasticsearch [4]. It features a user-friendly web interface for interacting with the data stored in the indexes and create dashboards, perform ad-hoc analyses, and visualise complex queries and aggregations. Additionally, data can also be extracted programmatically using Application Programming Interface (API) calls via a Python kernel in a JupyterHub environment for advanced data manipulation, cleaning, and analysis.

Apache NiFi is a data integration and processing tool used to extract data directly from EPR databases [5]. NiFi facilitates extract-transform-load (ETL) processes from diverse sources and transforms data to ingest into in an Elastic index. Unstructured EPR data within KCH was predominantly stored across multiple Microsoft SQL (MSSQL) tables, where Structured Query Language) SQL views have been developed to perform data transformation operations like joins and unions; Apache Nifi extracts the resulting data at different intervals depending on the data source and distribute data transformation tasks such as optical character recognition (OCR) for scanned documents and others across multiple servers for ingestion into Elastic indexes. Patient records are organised into episodes or visits, which are classified as inpatient, outpatient, emergency, and others. Changes in episode types can occur, such as when an emergency patient has an inpatient ward admission. Records are stored in Elastic indexes based on their respective types; examples include:

- EPR documents: Inpatient and outpatient notes and letters.
- Basic observations: Blood tests, scans, and pathology reports.
- Observations: Observed variables such as patients' weight, height, vital signs, and bed number.
- Orders: Diagnostic and therapeutic orders.
- Patient information management system (PIMS): Outpatient appointment data, including appointment date, time, modality, and cancellation reasons.
- Demographics: Maintains an up-to-date index of patient demographics.
- EPR inpatient: Maintains a list of all current inpatients across KCH.
- EPR allergies: Maintains a live list of every current and past allergy record.

### Comparison between Elastic and Smart Watchers

An Elastic Watcher is a highly customisable automated monitoring tool within Elasticsearch [6]. Elastic Watchers are particularly useful in clinical settings due to their versatility in executing queries and triggering near real-time email-based alerts based on predefined conditions. A watcher consists of a trigger that defines when the watcher checks for changes in the data, an input or data source, and an action, which determines what should happen when the watcher's conditions are met. In the context of this study, it is sending an email notification to a predefined list of recipients, with an attachment containing the desired clinical data.

Smart Watchers are an automated clinical alert system designed to monitor, extract, and process data from one or more Elastic indexes within a healthcare setting. Apache NiFi, Python scripts, and Elasticsearch are leveraged to generate near real-time alerts for specific clinical scenarios. Smart Watchers employ customisable data processing pipelines, created using Apache NiFi and Python scripts that are adaptable to specific clinical requirements. While Elastic Watchers and Smart Watchers are both suited for automated monitoring and near real-time alerting within healthcare settings, Smart Watchers offer a more flexible approach to querying and data manipulation. Elastic Watchers are primarily designed for data retrieval and not optimised for extensive data cleansing or preparation during the querying process. Despite Elastic Watchers being able to execute a series of queries in sequence and using the output of one query as input to the next query (via chain input), this is does not support querying across different indexes. In contrast, Smart Watchers leverage Python scripts to execute nested cross-index searches and extract data from external datasets. This allows for more complex data retrieval and analysis scenarios that extend beyond the capabilities of Elastic Watchers.

**Fig. 1:**
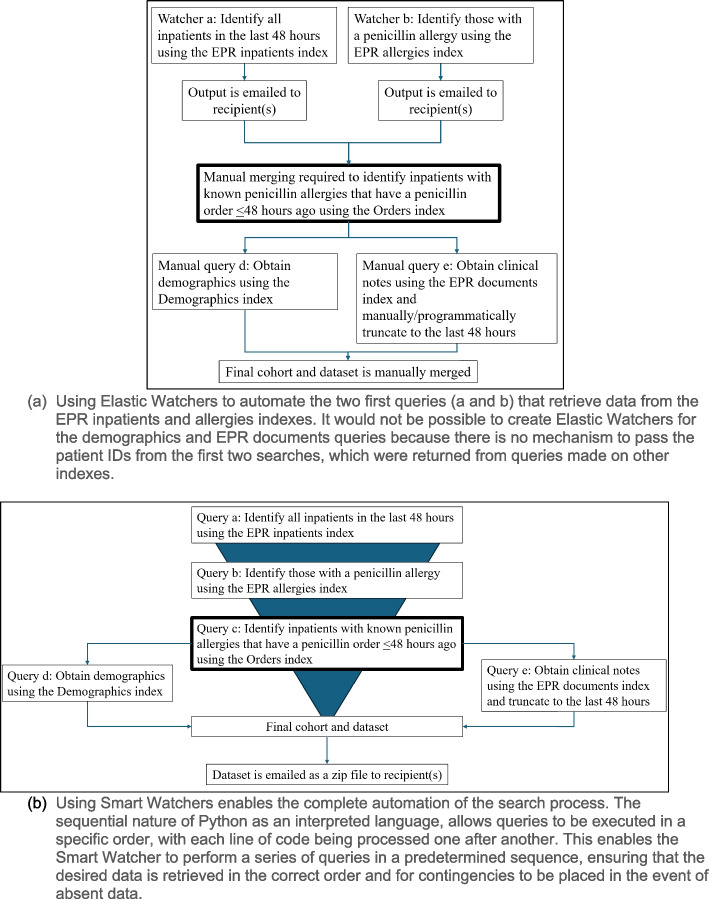
Comparison of search strategies for the penicillin delabeller project (see Table 1) using Elastic and Smart Watchers

For example, a recent project focused on identifying inpatients at KCH who had a documented penicillin allergy and a penicillin order within the last 48 h. The requesters required a daily extract of the target cohort, along with their demographics and clinical notes from the preceding 24 h only. This complex query required data retrieval and integration from EPR inpatients, EPR allergies, Orders, Demographics, and EPR documents indexes. Figure 1a illustrates the limitations of using Elastic Watchers, where two separate Watchers would need to be created to extract all inpatients in the last 48 h and all patients with penicillin allergies, resulting in two daily email notifications. The data would then require manual merging and querying to retrieve the relevant demographics and clinical notes. In contrast, Fig. 1b demonstrates the capabilities of Smart Watchers in completely automating the data retrieval process. Using Python scripts in an Apache NiFi processor group, Smart Watchers can execute all the queries in sequence, with queries D and E being performed in parallel to return the relevant demographics and clinical notes. The final dataset is stored in a ZIP file and attached to an email. The data can be sent as individual CSV files, a merged CSV or Excel file, or a specifically formatted CSV file tailored to the requester's needs. For instance, the data can be formatted for specific databases, such as REDCap (Research Electronic Data Capture), SQL or Microsoft Access. Furthermore, Python can be leveraged to clean and process data, or use regular expressions (regex) within the dataset to extract or filter data even further. Regex was applied in Query E, to the unstructured field from the EPR allergy output, to identify mentions of penicillin or symptoms of allergies. This helped prioritise more severe symptoms like 'anaphylaxis' over less specific ones like 'stomach upset'.

**Fig. 2:**
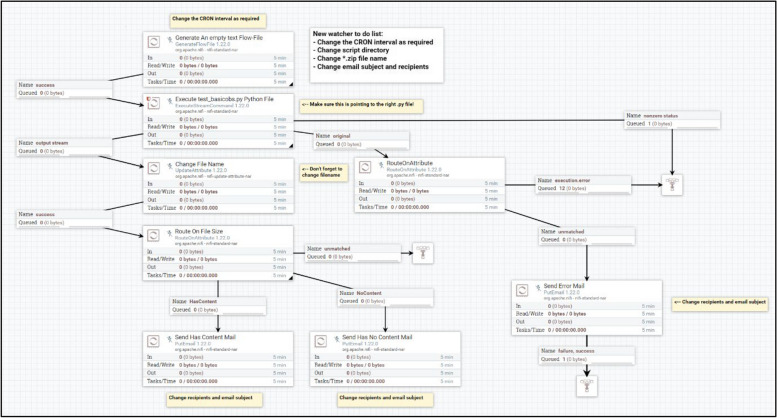
The default Smart Watcher Nifi processor group template. The Nifi processor group orchestrates a series of actions to manage data flow, extraction, and communication in a five-step process. Initially, a GenerateFlowFile processor, scheduled via Quartz-cron, creates an empty text file, which serves as a placeholder to initiate the data flow process and ensure that subsequent processors have a starting point. The empty text flow file is then managed by an ExecuteStreamCommand processor to execute a Python file that handles data queries, extractions, and transformations, storing results in an in-memory zip file to reduce server clutter, rather than storing the files on disk. Errors from the Python script or nonzero statuses trigger conditional routing by the RouteOnAttribute processor to a PutEmail processor, notifying the CogStack team via SMTP protocol for troubleshooting. Subsequently, an UpdateAttribute processor modifies the zip file name, and another RouteOnAttribute processor evaluates the zip file size to determine the routing path, ensuring relevant communication is sent to the requester and the CogStack team (who are only notified in the event when no data was found or an error has been encountered), either providing the extracted data or indicating no data was found, requiring further exploration

Both Apache Nifi and Elasticsearch are dockerised, with the latter deployed as part of a swarm across three servers. NiFi processor groups were built to automate data processing flows for each index and manage errors effectively, offer conditional routing capabilities, and send email notifications in the event of any data ingestion errors (an example of a Nifi processor group is illustrated in Fig. 2).

### Smart Watcher development process

**Fig. 3:**
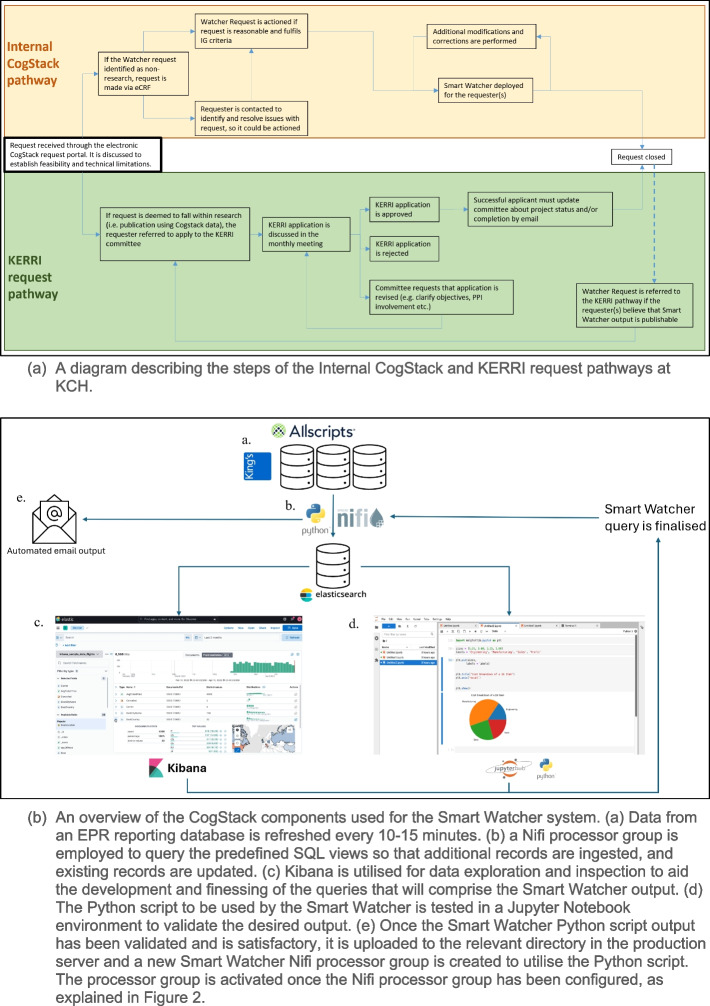
A diagram describing the steps of the request pathways to create Smart Watchers, and an overview of system components used for the Smart Watcher System

The CogStack team at KCH is responsible for development and maintenance of the CogStack analytical platform, enforcement of Information Governance (IG) standards, aiding requesters with their data requirements for audits, service evaluations, patient safety and research, which may involve Smart Watchers, depending on their requirements. The process of handling Smart Watcher requests begins when a requester, typically a KCH employee such as a clinician, PhD student, or researcher, submits a request to the CogStack team, as illustrated in Fig. 3a. Once deemed technically feasible and compliant with information governance, the request is approved. If the requested data from CogStack is to be published, then the request follows the King’s Electronic Records Research Interface (KERRI) pathway, where the requester must apply for KERRI committee approval. The KERRI committee, composed of clinicians, representatives of patients, and the KCH Caldicott Guardian, will discuss all submitted requests in their regular meetings. If the KERRI request is approved, or the requested data is to be used internally, as part of an audit, patient safety, or safety evaluation, then a member of the CogStack team will build the Smart Watcher. The Smart Watcher development process consists of the CogStack team member gathering requirements by liaising with the requester and using Kibana to inspect the data (see Fig. 3b). The indexes, fields, data types and outputs required for the query are identified. A Jupyter Notebook is used to prototype the Python script that will perform the queries and data manipulation. Once the Python script is finalised, it is uploaded to the Nifi directory, and a Nifi Smart Watcher processor group is created and configured for final deployment. Even after deployment, the Smart Watcher can be easily modified if an error is found. All Smart Watchers are logged and audited on a regular basis by the CogStack team to ensure compliance with information governance and security policies. Upon completion of the project in question, the Smart Watcher is deactivated.

### Example of a Smart Watcher

**Fig. 4:**
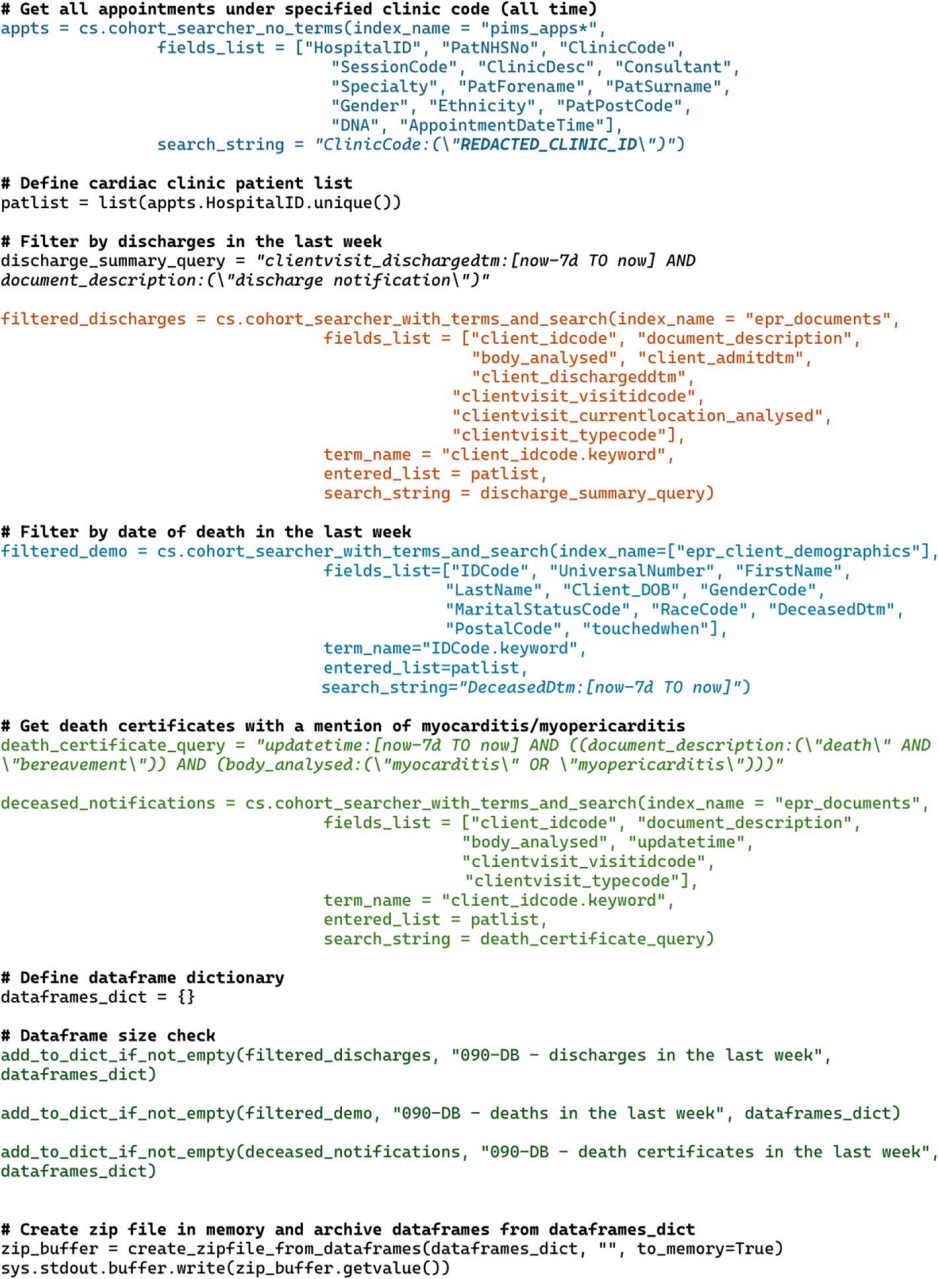
A sample of the Python code used to extract data for the Myocarditis admission/death tracker

The code below in Fig. 4 is an example of the Python script used in the Myocarditis admission/death tracker (see Table 1), where all Lucene syntax queries are in italic. The first query in blue identifies all the patients that attended the myocarditis clinic throughout all time, which identifies the cohort that is used in every subsequent query. The second query in red returns all discharge summaries in the last week for the myocarditis cohort, along with additional metadata such as latest location, episode number, admission and discharge time. The third query in purple identifies the time of death of any patients in the last 7 days and returns their demographics (name, date of birth, ethnicity and postcode). The fourth query in orange identifies any patients that have died in the last week with a mention of myocarditis in their death certificate. The Pandas Dataframes are saved as Microsoft Excel files as per the requester’s specifications and zipped in memory. This is passed as a Standard Out Nifi flow file. If the second, third and/or fourth query yielded no results, the empty dataset would be omitted in the dataset size check (highlighted in green). If there is no data at all, an empty zip file will be passed onto the next Nifi processor and the flowfile zero size would trigger an email to be sent informing the requester and the CogStack team that no data was found (see Fig. 2, under “Send Has No Context Email”). If an error is returned (such as a timeout or ingestion error), the error printout would be appended to the body of an email and be sent to the CogStack team for troubleshooting (see Fig. 2, under “Send Error Email”).

**Table 1:** An overview of watchers with their description, specialty, frequency, and complexity currently deployed by the CogStack team at KCH

Watcher name	Description	Complexity	Specialty	Watcher type	Frequency
ADAMTS13 tracker	Returns all ADAMTS13 test results and mentions in any EPR document for patients aged 16 to 50 years old	Low	Haematology	Audit	Weekly
Barrett’s oesophagus watcher	Returns all inpatients who had an OGD endoscopy report in the last week with mentions of Barrett’s oesophagus or gastric intestinal metaplasia from 2 specific wards	Low	Gastroenterology	Audit	Weekly
Cardiac MRI/Scleroderma watcher	Returns all cardiac MRI scan reports of patients who are known cases of scleroderma	Low	Cardiology	Audit	Weekly
COVID watcher	Returns all patients with a positive COVID PCR test result	Low	Respiratory	Audit	Daily
Diabetes Insipidus watcher	Returns all adult inpatients with mentions of diabetes insipidus in clinical note entries in the last 3 days and any desmopressin prescriptions. This cohort is filtered to only return those who have a high blood sodium level	Medium	Endocrinology	Research pilot	Daily
Inhaler technique auditor	Returns all inpatient inhaler orders across KCH	Low	Respiratory	Audit	Daily
IV Heparin infusion order tracker	Returns all inpatient IV heparin orders from several specified wards	Low	Haematology	Service improvement	Daily
Monkeypox watcher	Returns all patients with a positive monkeypox PCR test or mention in their EPR documents	Low	Virology	Audit	Daily
MRI enterogram tracker	Returns all MRI enterogram reports in the last week, along with patient demographics	Low	Gastroenterology	Audit	Weekly
Myocarditis admission/death tracker	Identifies all current myocarditis clinic attendees and from that cohort, returns those who have been discharged in the last week. It also identifies newly-published death certificates with mentions of myocarditis as the cause in the last week	Medium	Cardiology	Service improvement	Weekly
NICOR heart failure, myocardial infarction and myocarditis watcher	Retrieves demographics, admission data, clinical notes, comorbidities, imaging reports and specialist referrals, along with drug orders, blood test results and observations on admission and discharge for heart failure, myocardial infarction and myocarditis watcher	High	Cardiology	Research	Weekly
Out-of-hospital cardiac arrest tracker	Identifies all patients with a mention of out-of-hospital cardiac arrest in the last week	Medium	Cardiology	Research pilot	Weekly
Penicillin delabeller	Identifies current inpatients who are allergic to penicillin or other antibiotics, retrieves their admission data and identifiers and merges it into one spreadsheet. The pharmacist uses this list to visit the patient in-person and discuss potential of penicillin allergy delabelling	High	Microbiology	Research pilot	Daily
Pre-renal transplant checker	Returns all pre-renal transplant assessments in the last week, along with patient demographics	Low	Nephrology	Audit	Weekly
Rheumatology-MTX tracker	Identifies current Rheumatology clinic attendees and from that cohort, returns those who have passed away in the last 24 h or have abnormal liver enzymes and are currently on Methotrexate	High	Rheumatology	Service improvement	Daily
Sickle cell crisis watcher	Identifies all adult sickle cell inpatients who had a crisis mention (and crisis type) in the last week	Medium	Haematology	Service improvement	Weekly
VTE risk assessment in ED post take watcher	Identifies all emergency patients who had a VTE assessment containing the “High risk of VTE, low risk of bleeding” result	Low	Haematology	Service improvement	Daily
Warfarin order tracker	Returns all inpatient warfarin orders across KCH	Low	Haematology	Service improvement	Daily

A comparative time analysis was conducted to discern the difference between the time taken for manual data extraction using the EPR front-end and a Smart Watcher. The manual extraction process was initiated upon accessing a qualifying patient's data to quantify the time investment per specific data point from an identified patient and ensuring a fair comparison with the automated extraction process. Clinicians navigated to the relevant ward and manually extracted individual patient data, adhering to the defined aim(s) of the Smart Watcher as outlined in Table 1. The manual extraction process was inherently linear, investigating one patient at a time, while the Smart Watcher, executed data extraction across all matching patients within its operational timeframe. A scant number of data discrepancies issues arose involving three Smart Watchers shortly after deployment. The validation process revealed that missing patient data led to their misclassification, this was rectified by adding additional if-else statements to check the length of the resulting dataframes. If no data was present, then the script would skip that query and would inform the requester of the missing data. No additional issues were raised once those issues were rectified.

## Results

**Fig. 5:**
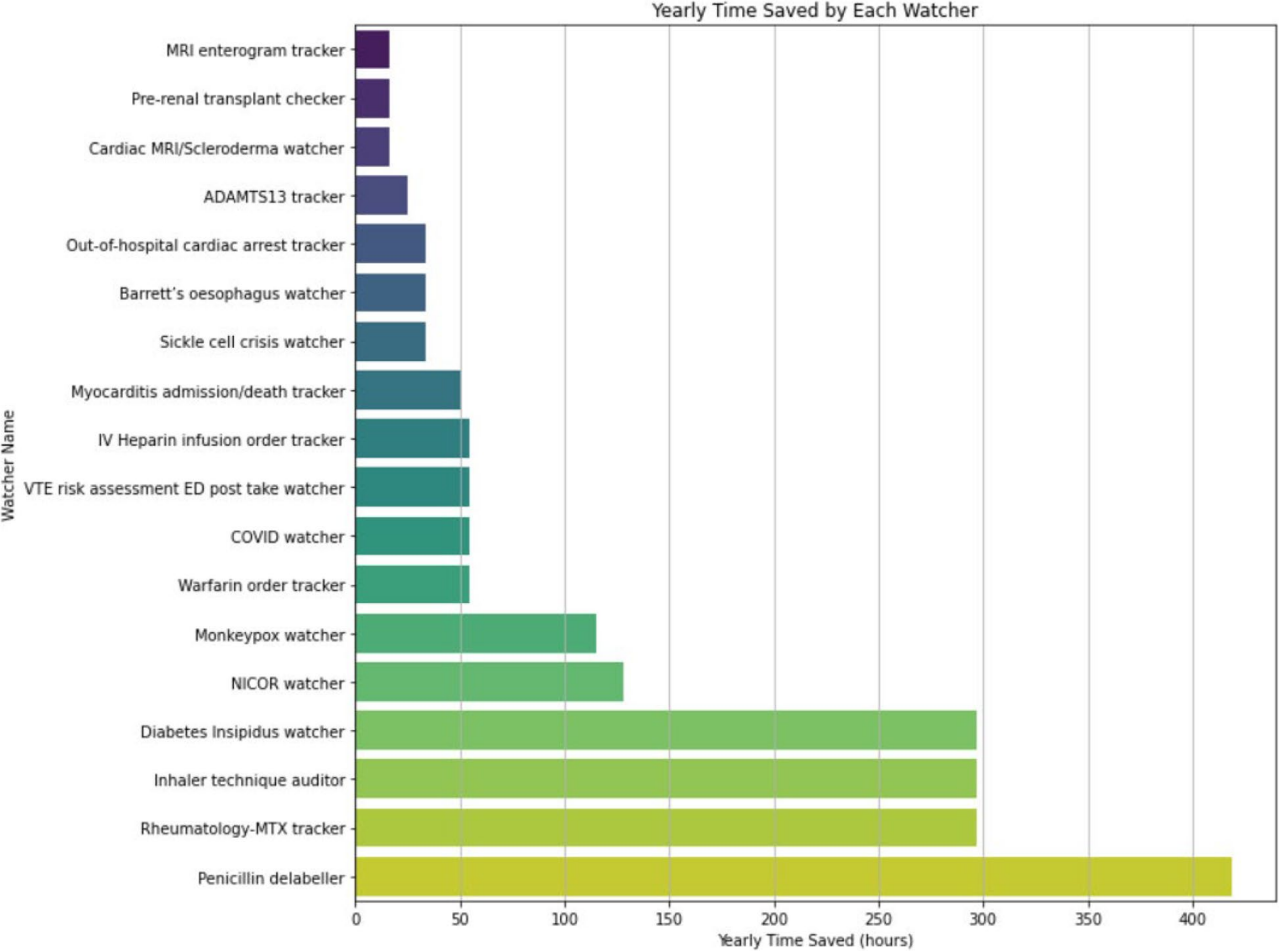
A bar chart illustrating the total time saved annually by using a Smart Watchers instead of manual data collection

Smart Watchers demonstrated a substantive reduction in manual effort, consistently saving between 90% and 98.67% of the required time for manual extraction methods, equating to a cumulative annual saving of 1,998.53 h across all Smart Watchers, as seen in Fig. 5:

Smart Watchers and manual extraction were not conducting identical tasks. Manual extraction performed by clinicians was localised to specific, identified patients, while Smart Watchers initiate their queries from all EPR patients and often exceeded the baseline of 10 patients, within a 1–2 min operational window. The theoretical financial savings afforded by Smart Watchers is notable; estimated savings amount to approximately £33,204.35 and £43,991.31 based on FY1 and ST1 hourly rates respectively, alternatively, this translates into an impactful 266.47 clinical workdays towards direct patient care [7].

## Discussion

The Smart Watcher system, built on Apache Nifi and Python, represents a significant advancement in clinical data monitoring and alerting. Its visual interface and complex data processing capabilities facilitate efficient extraction, transformation, and ingestion of EPR data from various sources. The inclusion of Python had enabled seamless integration with Nifi and efficient data processing from Elastic indexes and other sources, offering a more flexible approach compared to Elastic Watchers. A comprehensive literature review did not yield any clinical alert systems with comparable complexity and adaptability to the Smart Watcher system, particularly those that can perform data collection, cleansing, and preparation on the fly, such as using Python scripts within NiFi. This integration of advanced data processing directly into the alert system sets Smart Watchers apart, enabling it to handle a broader range of clinical data management tasks more efficiently than traditional systems. While Elastic Watchers represent the closest equivalent, it offers significantly fewer features and less flexibility in handling diverse clinical data types. Smart Watchers demonstrate notable enhancements in operational efficiency by concurrently querying data across EPR systems. This capability allowed for comprehensive dataset amalgamation within condensed timeframes and overcoming limitations of legacy systems. For instance, the Python-based splitter function enables precise term searches within specific time intervals from concatenated clinical notes, a feature not available in previous systems.

Despite its advantages, Smart Watcher deployment faces several challenges. Data inconsistencies and incomplete information can compromise accuracy, while poorly ingested documents, especially from OCR, may lead to missed data. Typos can introduce false positives or negatives, and temporal variations in data sources require tailored queries. To address these challenges, several strategies have been implemented, including wildcard and proximity searches, if-else statements for nested queries, and exclusion of non-Unicode characters before regex operations. However, some issues, such as mistyped terms and suboptimal OCR outputs, remain difficult to resolve due to the nature of real-world EPR data. The challenges encountered with Smart Watcher deployment closely align with those noted in a review by Blythe et al., who acknowledge the complexities and obstacles associated with implementing real-time clinical alert systems [8]. Like our findings, they emphasise the need for meticulous system management and continuous refinement, highlighting that overcoming these challenges is an ongoing process. Importantly, Blythe et al. concluded that clinical actions following alerts, such as immediate response and treatment protocols, played a crucial role in the effectiveness of such systems.

The impact of Smart Watchers on clinical practice and research has been substantial. The system has demonstrated significant time savings, estimated at 266.47 clinical workdays with theoretical financial savings ranging £33,204.35 to £43,991.31 per year based on FY1 and ST1 hourly rates. More importantly, Smart Watchers have been successfully applied in various clinical research scenarios, as demonstrated in Table 1. A study focusing on patients with CLL treated with ibrutinib used a Smart Watcher to identify a cohort of 76 patients, retrieving data on concurrent anticoagulant and antiplatelet agent usage, demographics, haematological diagnosis, ibrutinib exposure duration, and bleeding events [9]. In another application, a cardiac team employed a Smart Watcher to recruit 199 patients diagnosed with acute myocarditis. The system facilitated long-term cohort monitoring, leading to the conclusion that patients presenting with arrhythmias had a heightened risk of adverse events [10]. Smart Watchers were also integral in a retrospective observational study on Tumour Lysis Syndrome (TLS) in CLL patients, where the system's efficiency in cohort identification and data extraction revealed that risk category was the sole predictor of TLS events [11]. Furthermore, in a study on thromboprophylaxis prescriptions, Smart Watchers identified an adult patient cohort and retrieved vital treatment information, enabling analysis of prescription appropriateness based on local guidelines [12]. These examples highlight the immense value of Smart Watchers in improving patient pathways and clinical research efficiency. Without these automated systems, the manual identification and investigation of patient cohorts would be considerably more challenging and time-consuming, underscoring the transformational impact of this technology in advancing healthcare outcomes.

Ethical considerations have been carefully integrated into the Smart Watcher system. Smart Watcher data recipients are logged and must be approved by the involved lead clinician. Email accounts of recipients that depart from their role are automatically deactivated by the KCH ICT, which automatically prevents them from receiving any more emails. The CogStack team also reviews all active Smart Watchers and recipients quarterly and either remove inactive recipients or deactivate Smart Watchers that are no longer needed.

## Conclusion

The Smart Watcher system represents a significant stride forward in automated clinical data monitoring, alerting and processing. By leveraging Apache NiFi, Elasticsearch, and Python, it enables near real-time alerts for specific clinical scenarios, empowering healthcare professionals to identify patients at risk, prevent adverse events, and accelerate research efforts. Its integration with existing EPR systems streamlines workflows and enhances analytical efficiency. While challenges exist, particularly in data quality and system maintenance, the Smart Watcher system has demonstrated substantial potential in improving clinical practice and research. Its flexibility and extensibility open avenues for future enhancements, positioning it as a valuable tool for evidence-based decision-making and improved clinical outcomes.

### Future work

Future development of the Smart Watcher system should focus on several key areas, especially ingestion of ML model outputs, such as MedCAT annotations, into an Elastic index could enhance querying capabilities [13]. Developing methods to automatically accommodate changes in data sources would reduce the need for manual script modifications. Advanced OCR techniques should be investigated to improve the quality of ingested document data. To rigorously assess the system's impact, randomised control trials should be conducted to measure shifts in performance metrics such as alert response time and patient satisfaction before and after Smart Watcher implementation. User experience studies, including surveys and interviews would provide valuable insights into usability and efficiency among clinicians using the system. Additionally, retrospective case reviews would help evaluate the tangible impact of Smart Watchers on patient outcomes. These multifaceted approaches to evaluation and will help inform the development of additional features of future iterations of the Smart Watcher system. As the healthcare landscape continues to evolve, the ongoing development of systems like Smart Watchers will be critical in leveraging the wealth of available EPR data.

## Definitions

**Table:** 

Term Name	Definition
Apache Lucene	A high-performance, full-featured text search engine library.
Antiplatelet Drug	A type of medication that helps prevent platelets from clumping together to form a blood clot.
Anticoagulant Drug	A medication that helps prevent blood clots from forming, thereby reducing the risk of stroke, heart attack, and other serious conditions.
Caldicott Guardian	A senior person responsible for protecting the confidentiality of patient and service-user information and enabling appropriate information-sharing.
Dockerisation/Containerisation using Docker	A technique that utilizes Docker to ensure that applications run consistently in different computing environments.
Frontend	The part of a website or application that users interact with directly.
Ibrutinib	A medication used to treat certain cancers such as mantle cell lymphoma, chronic lymphocytic leukemia, and Waldenstrom's macroglobulinemia.
Information Governance	The management of information at an organization to support its regulatory, legal, risk, environmental, and operational requirements.
Jupyter Notebook	An open-source web application that allows you to create and share documents that contain live code, equations, visualizations, and narrative text.
Pandas	An open-source data analysis and manipulation tool built on top of the Python programming language.
Picture Archiving Systems	A medical imaging technology used to securely store, digitally transmit, and access images and reports.
Radiology Information Systems	A networked software system designed to manage medical imagery within a radiology department.
Thromboprophylaxis	Measures taken to prevent blood clots in veins, especially in patients who are at high risk, such as those undergoing surgery or with limited mobility.
Tumor Lysis Syndrome	A group of metabolic complications that can occur after treatment of cancer, typically characterized by high blood levels of potassium, phosphate, and uric acid.

## Data Availability

Project name: Enhancing Clinical Data Retrieval with Smart Watchers: A NiFi-based ETL Pipeline for Elasticsearch Queries. Project home page: https://github.com/CogStack/CogStack-NiFi Operating system(s): Ubuntu server v20 + Programming language: Python v3.7 + , Nifi v1.2 + Other requirements: Elasticsearch v8 + License: Elastic License 2.0 Any restrictions to use by non-academics: Not applicable.

## Abbreviations

API: Application programming interface
CLL: Chronic lymphocytic leukemia
Dataframe: Pandas dataframe
EPR: Electronic patient record
ETL: Extract, transform, load
FY1 Doctor: Foundation year 1 doctor
KCH: King's college hospital NHS foundation trust
KERRI: King's electronic records research interface ethics committee
ML model: Machine learning model
MSSQL: Microsoft SQL server
NiFi: Apache NiFi
NLP: Natural language processing
OCR: Optical character recognition
PIMS: Patient information management system
Regex: Regular expressions
SQL: Structured query language
ST1 Doctor: Specialist trainee year 1 doctor
